# Fit for the Next Fifty Exercise Program: 25 Years of Reflections, Results, and Partnerships

**DOI:** 10.1093/geroni/igab046.3253

**Published:** 2021-12-17

**Authors:** Mary Pagan

**Affiliations:** SUNY Oswego, Baldwinsville, New York, United States

## Abstract

Consistent exercise provides a multitude of physical, social, and emotional benefits. Common barriers to regular exercise for older adults include time, transportation, risk of injury, existing limitations, and negative experiences or attitudes about exercise. Fit for the Next Fifty is a comprehensive exercise and wellness program designed to address barriers and excuses. The program , based in CNY, has an impressive 25 year history of providing a unique mix of aerobic, strength training, yoga, and balance-based ballet. Participants (100-120) attend up to 5 classes per week at no charge during summer months and continue through winter months for a small fee. Developing and sustaining funding partnerships has been critical to the long-term success of Fit for the Next Fifty. Participants, ages 60-96, are active providers of feedback and suggestions, a key component to keeping the music, movements, and fellowship enjoyable and meaningful for over two decades. Program details, participant pictures and testimonials, research results, surveys across the years, and partnering/funding strategies provided. Of special interest is the social support dimension of the program. Participants have developed a sophisticated network to support each other outside of the exercise and wellness programs provided by Fit for the Next Fifty.

